# Evaluation of Surveillance Methods for Staphylococcal Toxic Shock Syndrome

**DOI:** 10.3201/eid1505.080826

**Published:** 2009-05

**Authors:** Lindsey Lesher, Aaron DeVries, Richard Danila, Ruth Lynfield

**Affiliations:** Minnesota Department of Health, St. Paul, Minnesota, USA (L. Lesher, A. DeVries, R. Danila, R. Lynfield); University of Minnesota, Minneapolis, Minnesota, USA (A. DeVries)

**Keywords:** Staphylococci, bacteria, epidemiology, Staphylococcus aureus, toxic shock syndrome, surveillance, dispatch

## Abstract

We compared passive surveillance and International Classification of Diseases, 9th Revision, codes for completeness of staphylococcal toxic shock syndrome (TSS) surveillance in the Minneapolis–St. Paul area, Minnesota, USA. TSS-specific codes identified 55% of cases compared with 30% by passive surveillance and were more sensitive (p = 0.0005, McNemar χ^2^ 12.25).

Staphylococcal toxic shock syndrome (TSS) is a severe illness associated with toxin-producing *Staphylococcus aureus*. First named in 1978, TSS has been associated with tampon use, intravaginal contraceptive devices, and skin infections, particularly after surgical procedures (*1**,**2*). In January 1980, the Minnesota Department of Health (MDH) initiated surveillance for TSS with active and passive components. The national incidence of TSS decreased during 1980–1996 (*3**,**4*) after removal of high-absorbency tampons from the market and public awareness campaigns. In subsequent years, surveillance methods in Minnesota were changed to a solely passive surveillance system that relied on clinicians to report cases. MDH uses the 1997 case definition of the Centers for Disease Control and Prevention (CDC) (Atlanta, GA, USA) to determine case criteria for any probable TSS case (>4 clinical criteria and laboratory criteria) considered reportable (Table 1) (*5*). Given the complexity of the case definition, we suspected that TSS underreporting was likely.

**Table 1:** Staphylococcal toxic shock syndrome case definitions*

Criteria	Definition
Clinical	
Fever	Temperature >38.9°C (102.0°F)
Rash	Diffuse macular erythroderma
Desquamation	1–2 weeks after onset of illness, particularly on the palms and soles
Hypotension	Systolic blood pressure <90 mm Hg for adults or <5th percentile by age for children <16 years of age; orthostatic decrease in diastloc blood pressure >5 mm Hg from lying to sitting, orthostatic syncope, or orthostatic dizziness
Multisystem organ involvement†	
Gastrointestinal	Vomiting or diarrhea at onset of illness
Muscular	Severe myalgia or creatine phosphokinase level at least twice the upper limit of normal
Mucous membrane	Vaginal, oropharyngeal, or conjunctival hyperemia
Renal	Blood urea nitrogen or creatinine at least twice the upper limit of normal for laboratory or urinary sediment with pyuria (>5 leukocytes by high-power field) in the absence of urinary tract infection
Hepatic	Total bilirubin, alanine aminotransferase, or aspartate aminotransferase levels at least twice the upper limit of normal
Hematologic	Platelet counts <100 × 10^9^/L
Central nervous system	Disorientation or alterations in consciousness with focal neurologic signs when fever and hypotension are absent
Laboratory	
Culture	If obtained, negative results on blood, throat, or cerebrospinal fluid cultures (blood culture may be positive for *Staphylococcus aureus*)
Titer	If obtained, no increase in titer for Rocky Mountain spotted fever, leptospirosis, or measles
Case classification	
Probable	Meets laboratory criteria and in which 4 of 5 clinical findings described above are present
Confirmed	Meets laboratory criteria and in which all 5 of the clinical findings described above are present, including desquamation, unless the patient dies before desquamation occurs

*From the US Centers for Disease Control and Prevention (*5*). †Involving >3 organ systems.

Recently, several factors, including increasing prevalence of community-associated methicillin-resistant *S*. *aureus* that carries superantigens and the trend toward earlier menarche, suggested that the incidence of TSS might be increasing (*6**,**7*). Additionally, the number of requests for superantigen testing in the Minneapolis–St. Paul (MSP) area made to a reference microbiology laboratory that tests staphylococcal isolates from TSS cases increased during 2000–2003 (*8*). To determine the incidence of TSS, active surveillance was initiated at all MSP area hospitals using International Classification of Diseases, 9th Revision (ICD-9), codes assigned at hospital discharge. We compared passive surveillance reports with ICD-9 codes to determine an effective and efficient surveillance method for TSS.

## The Study

The MSP area is composed of 7 counties with a population of 2,642,056 (2000 US Census) and 24 acute-care hospitals. Requests were sent to medical record departments of these hospitals for data on inpatients discharged from hospitals from January 1, 2000, through December 31, 2003, whose medical records indicated >1 of the select ICD-9 study codes. Medical records from all hospitalizations receiving the TSS-specific code (040.82 or 040.89) were reviewed (Figure), and a 20% random sample of medical records from hospitalizations that received >1 nonspecific TSS study code (Table 2) from within each hospital was reviewed. Each medical record was reviewed for TSS case criteria and pertinent epidemiologic and clinical information. Additionally, death certificates assigned the ICD-10 code for TSS (A48.3) and cases from the Minnesota Unexplained Critical Illness and Death of Possible Infectious Etiology project (UNEX) (*9*) during 2000–2003 were reviewed. TSS cases identified through ICD-9 code searches were compared with cases reported to MDH during 2000–2003. Data were analyzed with Stata version 9 (StataCorp, College Station, TX, USA). Statistical analyses included Pearson and McNemar χ^2^ tests.

**Figure:**
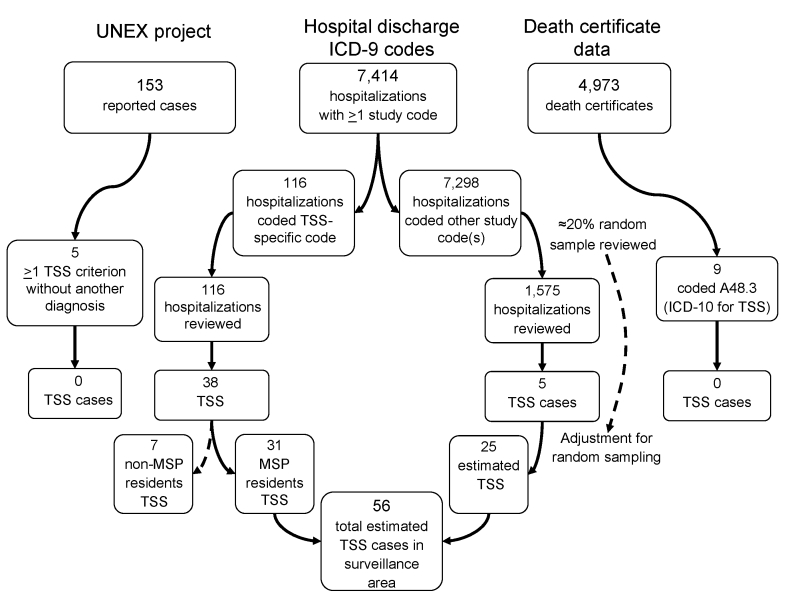
Flow diagram of toxic shock syndrome (TSS) case ascertainment. TSS cases were identified from International Classification of Diseases, 9th Revision (ICD-9), codes assigned at hospital discharge, cases reported to the Minnesota Unexplained Critical Illness and Death of Possible Infectious Etiology project (UNEX), and death certificate data by using International Classification of Diseases, 10th Revision (ICD-10), code A48.3. MSP, Minneapolis–St. Paul area.

**Table 2:** ICD-9 study codes used for staphylococcal toxic shock syndrome case ascertainment*

Code	Associated diagnosis
Specific toxic shock syndrome code	
040.89 or 040.82†	Toxic shock syndrome
Nonspecific toxic shock syndrome codes	
038.11	*Staphylococcus aureus* septicemia
038.19‡	Other staphylococcal septicemia
038.9	Unspecified sepsis
785.50	Shock without mention of trauma
785.59 or 785.52†	Sepsis

*ICD-9, International Classification of Diseases, 9th Revision. †The code assigned to toxic shock syndrome changed from 040.89 to 040.82 on October 1, 2002, and the code assigned to sepsis changed from 785.59 to 785.52 on October 1, 2003. Although the codes changed, their associated diagnoses remained unchanged and are considered mutually exclusive. ‡Code eliminated after interim analysis of 627 medical records at 14 of 24 hospitals. Of 122 records receiving only this code, 40% had 0 case criteria, all had <3 criteria, and 88% had bacteremia with a staphylococcal species other than *S. aureus*.

Of 7,414 hospitalizations with >1 study code, 116 (1.6%) were assigned the TSS-specific code and were reviewed (Figure). Of the remaining 7,298 hospitalizations assigned >1 nonspecific TSS code, 1,575 (21.6%) randomly selected hospitalizations were reviewed. Of these 1,691 hospitalizations, 55 had 5 or 6 criteria for TSS, of which 12 (22%) met the CDC case definition for streptococcal TSS, and 7 were non-MSP residents. The remaining 36 cases were probable or confirmed TSS. No cases from UNEX or death certificate searches met the TSS case definition. Of the 36 TSS cases, 17 (47%) were reported to MDH by passive surveillance. Thirty-one (86%) cases were found by using TSS-specific ICD-9 codes. Five cases were found by using non-TSS–specific ICD-9 codes. After adjusting for 20% random sampling for cases identified by non-TSS–specific codes, we identified the estimated number of cases by using non-TSS–specific codes to be 25. This analysis resulted in 56 estimated TSS cases identified in the surveillance area during 2000–2003 by using ICD-9 codes.

The TSS-specific ICD-9 code search identified 31 of the 56 estimated TSS cases (sensitivity 55%, specificity 99%). Seventeen cases were reported to MDH by passive surveillance (sensitivity 30%, specificity 99.9%); all were coded with the TSS-specific code. The TSS-specific ICD-9 code search was more sensitive than passive surveillance (p = 0.0005, McNemar χ^2^ 12.25). Of those cases reported to MDH, more were likely to be associated with menstruation (14/17 vs. 5/19; p<0.001) and to have had a positive test result for *S*. *aureus* (16/17 vs. 11/19; p = 0.01). Twenty-seven of 36 TSS cases detected had a bacterial culture positive for *S*. *aureus*. The 3 TSS cases with methicillin-resistant *S*. *aureus* isolates were not reported to MDH. The positive predictive value of being a case among those coded with the TSS-specific code was 27% (31/116). In 68 of 116 cases that received the TSS-specific code, there was clinical suspicion of TSS, but these cases did not meet the clinical case definition (<5 criteria): 10 were streptococcal TSS and 7 were in non-MSP residents. All 17 cases reported to MDH were detected through the ICD-9 code search.

## Conclusions

Surveillance for TSS is challenging given the lack of a diagnostic test and a case definition with multiple components. Under the current passive surveillance system, between one third and half of potential TSS cases were identified. Discrepancies were found in reporting, with menstruation-associated cases more likely to be reported to MDH than nonmenstrual-associated cases. This discrepancy was observed with prior active surveillance efforts (*10*).

Using ICD-9 codes, we found 12 TSS cases that were of streptococcal etiology. Accuracy may be improved by developing separate ICD-9 codes specific for staphylococcal, streptococcal, or unidentified TSS. In addition to the TSS-specific ICD-9 code, we selected 5 other ICD-9 codes on the basis of previous studies to address the concern that TSS cases may be classified under a staphylococcal infection or sepsis code, but not the TSS-specific code (*8**–**10*). These 5 additional non-TSS–specific ICD-9 codes required reviewing 1,575 medical records; only 5 (0.3%) additional TSS cases were identified. The non-TSS–specific ICD-9 codes detected 25 estimated cases. However, this detection required 8 trained staff and substantial resources with ≈40 minutes required per medical record review.

Passive surveillance requires fewer public health resources because it relies on clinicians to report cases. Active surveillance involves public health resources in identifying cases. The disadvantage of passive surveillance is the potential for missed cases. Despite possible inaccuracies associated with the assignment of ICD-9 codes, these codes represent a standardized data source that may be readily available. In the absence of a specific diagnostic test, ICD-9 codes represent an efficient method for surveillance and following trends.

Medical record abstraction per hospitalization was labor- and resource-intensive and is not feasible for most health departments. With increasing use of automated electronic reporting for disease surveillance (*11*), querying hospital discharge data for the TSS-specific ICD-9 code is a feasible adjunct to passive surveillance to detect TSS trends over time. Consequently, it is imperative that clinicians and coders are thorough to ensure that ICD-9 codes are accurate.

We found it useful to add regular ICD-9 code searches for TSS-specific codes as an active surveillance adjunct to our passive surveillance system. This addition increases sensitivity of TSS surveillance with a minimal increase in resources. Use of this more sensitive system increases the ability to detect trends in TSS, which may develop because of changes in bacterial virulence characteristics, host characteristics such as the use of new devices or products, changes in human behavior, or changes in host susceptibility. Evaluation of this approach in other areas to assess sensitivity of TSS surveillance would be useful because coding practices may differ.

## References

[1] Reingold AL, Hargrett NT, Dan BB, Shands KN, Strickland BY, Broome CV. Nonmenstrual toxic shock syndrome: a review of 130 cases. Ann Intern Med. 1982;96:871–4. 7091959 10.7326/0003-4819-96-6-871

[2] Kain KC, Schulzer M, Chow AW. Clinical spectrum of nonmenstrual toxic shock syndrome (TSS): comparison with menstrual TSS by multivariate discriminant analyses. Clin Infect Dis. 1993;16:100–6. 8448283 10.1093/clinids/16.1.100

[3] Gaventa S, Reingold AL, Hightower AW, Broome CV, Schwartz B, Hoppe C, Active surveillance for toxic shock syndrome in the United States, 1986. Rev Infect Dis. 1989;11(Suppl 1):S28–34. 2928646 10.1093/clinids/11.supplement_1.s28

[4] Hajjeh RA, Reingold A, Weil A, Shutt K, Schuchat A, Perkins BA. Toxic shock syndrome in the United States: surveillance update, 1979–1996. Emerg Infect Dis. 1999;5:807–10. 10603216 10.3201/eid0506.990611 PMC2640799

[5] Centers for Disease Control and Prevention. Toxic shock syndrome 1997 case definition [cited 2005 Oct 5]. Available from http://www.cdc.gov/epo/dphsi/casedef/toxicsscurrent.htm

[6] McDowell MA, Drody DJ, Hughes JP. Has age at menarche changed? Results from the National Health and Nutrition Examination Survey (NHANES) 1999–2004. J Adolesc Health. 2007;40:227–31. 10.1016/j.jadohealth.2006.10.002 17321422

[7] Euling SY, Herman-Giddens ME, Lee PA, Selevan SG, Juul A, Sørensen TI, Examination of US puberty-timing data from 1940 to 1994 for secular trends: panel findings. Pediatrics. 2008;121:S172–91. 10.1542/peds.2007-1813D 18245511

[8] Schlievert PM, Tripp TJ, Peterson ML. Reemergence of staphylococcal toxic shock syndrome in Minneapolis–St. Paul, Minnesota, during the 2000–2003 surveillance period. J Clin Microbiol. 2004;42:2875–6. 10.1128/JCM.42.6.2875-2876.2004 15184497 PMC427823

[9] Hajjeh RA, Relman D, Cieslak PR, Sofair AN, Passaro D, Flood J, Surveillance for unexplained deaths and critical illnesses due to possibly infectious causes, United States, 1995–1998. Emerg Infect Dis. 2002;8:145–53. 11897065 10.3201/eid0802.010165 PMC2732455

[10] Osterholm MT, Forfang JC. Toxic-shock syndrome in Minnesota: results of an active-passive surveillance system. J Infect Dis. 1982;145:458–64. 7069226 10.1093/infdis/145.4.458

[11] Lazarus R, Klompas M, Campion FX, McNabb SJ, Hou X, Daniel J, Electronic support for public health: validated case finding and reporting of notifiable diseases using electronic medical data. J Am Med Inform Assoc. 2009;16:18–24. 10.1197/jamia.M2848 18952940 PMC2605594

